# Age of Hypertension Onset: Overview of Research and How to Apply in Practice

**DOI:** 10.1007/s11906-020-01071-z

**Published:** 2020-08-27

**Authors:** Karri Suvila, Ville Langén, Susan Cheng, Teemu J. Niiranen

**Affiliations:** 1 Division of Medicine, Turku University Hospital, Turku, Finland; 2 Department of Internal Medicine, University of Turku, Turku, Finland; 3 Department of Geriatrics, University of Turku, Turku, Finland; 4 The Framingham Heart Study, Framingham, MA USA; 5 Barbra Streisand Women’s Heart Center, Smidt Heart Institute, Cedars-Sinai Medical Center, Los Angeles, CA USA; 6 Department of Public Health Solutions, Finnish Institute for Health and Welfare, Turku, Finland

**Keywords:** Blood pressure, Hypertension, Age of hypertension onset, Hypertension heritability, Hypertension and cardiovascular disease, Clinical implications

## Abstract

**Purpose of Review:**

To review the current evidence on research related to age of hypertension onset—its definition, correlates, heritability, and association with adverse outcomes. We also propose a framework for implementing assessment of hypertension onset age into clinical practice.

**Recent Findings:**

Prior studies have used both objective measurements and self-report to determine age of hypertension onset or early-onset hypertension. Yet, no criterion for standard definition currently exists for either. Data from epidemiological and clinical studies demonstrate that early-onset hypertension is a highly heritable trait that confers an increased risk for cardiovascular death and end-organ damage compared with late-onset hypertension.

**Summary:**

Literature to date suggests that (parental) age of hypertension onset can be feasibly assessed for estimating (1) risk of future hypertension in non-hypertensive persons; and (2) the propensity for cardiovascular disease in individuals with established hypertension.

## Introduction

Elevated blood pressure (BP) affects over 1 billion people globally and is known to be highly age-dependent [1–5]. The patterns of BP progression over age in the general population are well-documented. On average, systolic BP (SBP) rises throughout life in most individuals and finally reaches a plateau in late life [6]. However, BP trajectories over age can vary and are likely related to different levels of cardiovascular risk [7, 8]. Particularly, younger hypertensive individuals, who are more likely to be undiagnosed and undertreated than older patients, are at high risk of lifetime cardiovascular disease [9]. Despite chronological age being the strongest risk factor for both hypertension and cardiovascular disease, limited focus has been given until recently to age of hypertension onset as a potential risk factor in patients.

Several prior studies have examined age of disease onset and its impact on adverse outcomes in the context of chronic diseases, such as diabetes and obesity [10–13]. These studies have concluded that early disease onset usually results in a considerably poorer prognosis than late onset. Despite the similarities between hypertension, diabetes, and obesity as chronic disease states, the prognosis and clinical relevance of hypertension that *begins* in early versus late life have remained mainly unknown. Previous studies have introduced several indices for assessing an individual’s long-term BP exposure, such as time-averaged BP, cumulative BP, and BP trajectory patterns [14••, 15–17]. In all of these studies, long-term BP exposure was more closely associated with adverse outcomes than single BP measurements. However, these indices usually require complex calculations and availability of a large number of historical BP recordings, limiting their implementation into everyday clinical practice. Thus, more feasible risk stratification methods are called for to better estimate the long-term lifetime exposure to high BP in hypertensive patients.

The current hypertension treatment guidelines do not consider age of hypertension onset in the management of hypertensive individuals [18, 19]. However, a growing literature suggests that it may be important to distinguish between early-onset and late-onset hypertension as a familial trait when assessing an individual’s risk for hypertension, and as a specific type of BP trait when estimating risk for cardiovascular outcomes in individuals with established hypertension. This review will summarize current evidence on research related to age of hypertension onset—its definition, correlates, heritability, and association with adverse outcomes. We also propose a framework for implementing assessment of hypertension onset age into clinical practice.

## Definition of Hypertension Onset Age

Three methods have been used in prior epidemiological and clinical studies to derive information on the age at which an individual’s BP level meets the criteria for hypertension for the first time: serial BP measurements, medical records, or self-report. Objective BP measurements have been the most commonly used method in epidemiological studies, such as the Framingham Heart Study or the Johns Hopkins Precursors Study. In these studies, standardized, repeated BP measurements performed at regular intervals are usually available, making it possible to objectively define the time and age of hypertension onset [8, 20•, 21••, 22, 23•]. In several of these studies, hypertension was defined as SBP/diastolic BP (DBP) ≥ 140/90 mmHg or use of antihypertensive medication at two or more consecutively attended examinations. This definition has been used for to reduce variation and to represent a more durable change in BP [8, 21••, 22, 23•]. However, in other studies, only one instance of elevated BP was required for hypertension onset [20•].

Apart from epidemiological studies, some cross-sectional observational studies have also used documented BP reports or medical records for determining hypertension onset age [20•, 24]. In contrast, most clinical studies, in which long-term BP data are not usually available, have defined hypertension onset using self-reported age at when the patient was first diagnosed or treated for hypertension [20•, 24, 25•, 26, 27, 28••, 29]. The latter two methods for defining hypertension onset (medical records and self-report) are markedly different from the former (objective BP measurements). Information from medical records and self-report mainly reflects the age of diagnosis instead of the true age of onset derived from repeated, objective measurements. A second major difference between these two methods is that self-reported age of hypertension onset is always dependent on the then-prevailing definition of hypertension, which has changed from > 180/110 to > 130/80 mmHg over the past seven decades. Objective measurements are therefore likely to be the most accurate method for defining age of hypertension onset and should be used for research purposes. However, hypertension onset age based on self-report has a strong correlation with objectively defined onset age [29] and thereby provides a convenient and practical alternative for clinical practice, where repeated BP measurements across several decades are not usually easily available.

In addition to the diverse methods used for defining age of hypertension onset, studies have also used varying age thresholds for early-onset hypertension. In several reports, early-onset hypertension has been defined as onset at age ≤ 55 years [8, 20•, 21••, 23•]. This age threshold is in accordance with the British hypertension guidelines, which recommend different first-line treatment for patients over and under 55 years of age [30]. In other studies, the participants have been divided into several 10-year age of onset categories mainly based on convenience, i.e., depending on the age range of the study sample [22, 25•, 28••, 29, 31]. In any case, no criterion for standard definitions currently exists for either age of hypertension onset or early-onset hypertension. Additional research is therefore needed on the agreement between age of hypertension onset defined by using different methods. In addition, universal definitions for early-onset hypertension and age of hypertension onset are needed for both research and clinical purposes.

## Correlates of Hypertension Onset Age

Only three small studies have assessed the clinical characteristics related to early-onset hypertension in men and women [26, 32, 33]. The first study examined 82 Taiwanese patients who developed hypertension before the age of 40 years [32]. The majority of these patients (56 out of 82) were male and had consistently higher triglyceride levels compared with controls. In addition, male patients had higher body mass index, whereas women had higher uric acid, suggesting gender-specific differences in the correlates of early-onset hypertension. In the second study [33], only nine Japanese students with early-onset hypertension were identified after screening thousands of subjects < 30 years of age at annual university student check-ups. All of these patients were men, and eight out of nine had parents who were treated with antihypertensives, reflecting the genetic predisposition to early-onset hypertension. In an additional third study with no men included in the study sample, women with a hypertensive disorder of pregnancy reported diagnosis of hypertension 7.7 years earlier than women without pregnancy complications [26]. Despite these studies being restricted by their small study samples, at least male sex, higher body mass index, and genetic background appear to be key correlates of early-onset hypertension. Nevertheless, once early-onset hypertension has been established, healthcare providers need to investigate these young patients for secondary causes of hypertension, as recommended by the current guidelines [18, 19]. However, one study has suggested that the prevalence of secondary hypertension could surprisingly be even lower in younger than in older patients [34]. In conclusion, despite these preliminary results, the data are currently too limited to make any robust recommendations on which individuals should be screened for early-onset hypertension in clinical practice.

## Age of Hypertension Onset and Hypertension Heritability

**Fig. 1:**
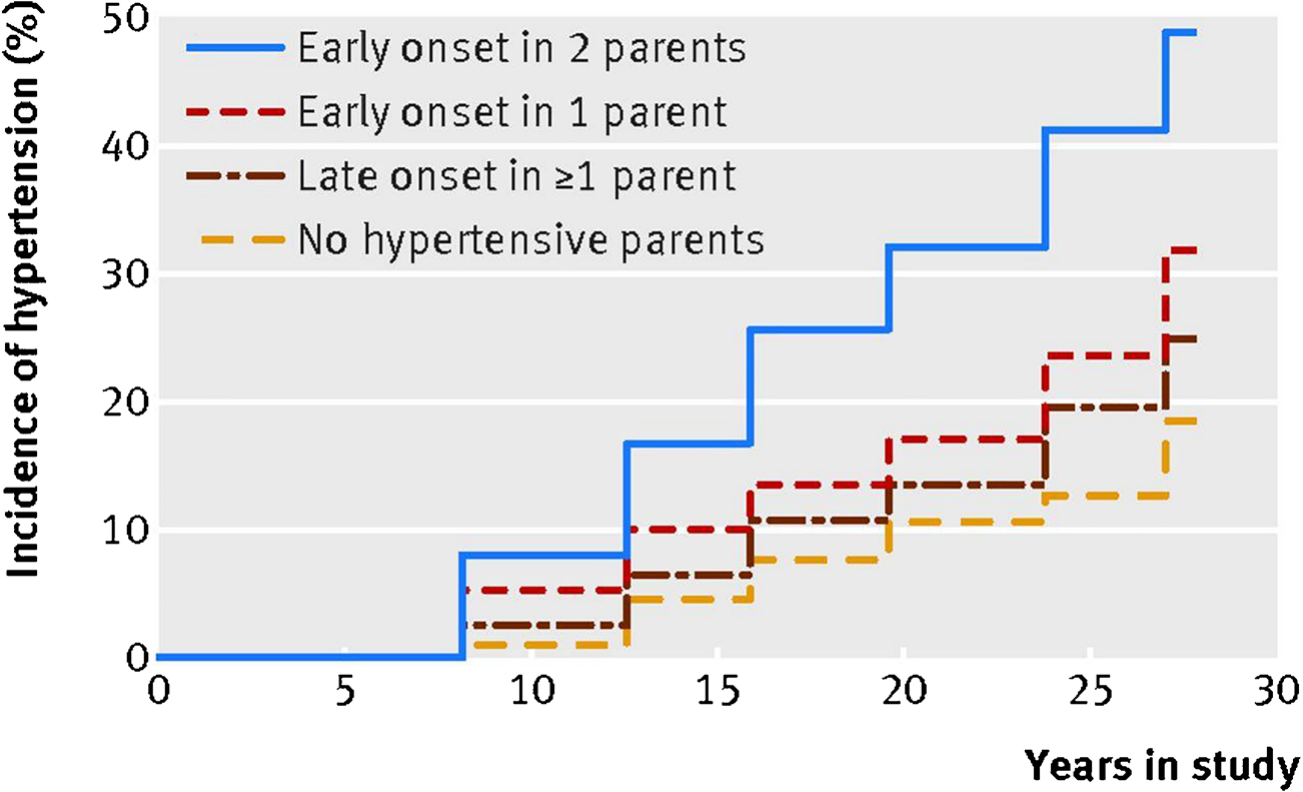
Cumulative incidence of hypertension in relation to parental age of hypertension onset (from BMJ 2017;357:j1949). Published with the permission of BMJ Publishing Group Ltd.

Several studies have demonstrated that hypertension onset age is a highly heritable trait. The cumulative incidence of hypertension by parental hypertension status in the Framingham Heart Study Offspring Cohort is shown in Fig. 1 [21••]. In the Framingham and Johns Hopkins Precursors Studies, early-onset hypertension (onset at age ≤ 55 years) in both parents was associated with over 3.4- and 6.2-fold adjusted risks of hypertension in offspring, respectively [20•, 21••]. In contrast, late-onset parental hypertension in both parents, however, carried only non-significant or 1.5-fold risks of hypertension in both studies. Additionally, this heritability effect seems to cross over generations, as early-onset hypertension in grandparents has been demonstrated to predict hypertension also in grandchildren [23•]. Namely, in 3608 third-generation participants of the Framingham Heart Study, risk for hypertension was conferred simultaneously by presence of early-onset hypertension in parents (odds ratio of 2.10) as well as in grandparents (odds ratio of 1.33). These results suggest that a considerable familial susceptibility for hypertension exists, and this predisposition could even cross generations. However, further studies are required to shed light on the mechanisms underlying transgenerational risk for hypertension and its clinical implications.

Besides population-based studies, early-onset hypertension has been associated with various single nucleotide polymorphisms in genomic studies [27, 35–41]. However, no distinct genetic variants or genes have been identified to specifically and consistently link with early-onset hypertension across different study populations. Based on the current evidence, the association between genetic variation and early hypertension onset age seems to vary highly by geographic and ethnic backgrounds [35–38]. As the precise genetic etiology of early-onset hypertension still remains elusive, additional research in this domain, and particularly on the impact of polygenic risk scores on the age of hypertension onset, is needed.

## Age of Hypertension Onset and Disease Risk

Even though hypertension is a commonly known risk factor for myocardial infarction and stroke [42], limited evidence exists on the effects of hypertension onset age on cardiovascular disease risk. Only three previous studies to date have assessed the relation between hypertension onset age and various cardiovascular disease outcomes ([21••, 22, 28••]; Table 1). One of these studies examined the association between hypertension onset age and hypertension-mediated organ damage in 2680 middle-aged participants of the Coronary Artery Risk Development in Young Adults (CARDIA) study [22]. Two studies that were performed in primary care patients (*n* = 10,313) or in Framingham Heart Study participants (*n* = 3614) used cardiovascular death and other cardiovascular disease events as the outcome of interest [21••, 28••]. Overall, the results from all these studies consistently demonstrate that hypertension onset at an earlier age, without any clear threshold, is a potent risk factor for both subclinical and subsequent overt cardiovascular disease (Table 1). In addition, the odds of subclinical disease seem to be similar irrespective of whether the age of onset is based on self-report or objective measurements [22, 29]. The risk estimates reported in these studies remained highly significant even after adjusting for other cardiovascular risk factors. However, the study conducted by Buck et al. in the 1980s used only DBP for diagnosing hypertension, and the results were not adjusted for all relevant confounders, such as cholesterol or glucose levels [28••].

**Table 1:** Age of hypertension onset and odds of adverse outcomes

Study	N	Outcome	Hypertension onset age (years)	Odds ratio (95% CI)	*p* value
Buck et al. [28••]	10,313	CVD event^*^	40–49	5.2 (n/a)	n/a
			50–59	1.8 (n/a)	n/a
			60–65	1.2 (n/a)	n/a
			No hypertension	Ref.	
Niiranen et al. [21••]	3614	CVD death	< 45	2.19 (1.77–2.70)	< 0.001
			45–54	2.10 (1.67–2.63)	n/a
			55–64	1.86 (1.48–2.34)	n/a
			≥ 65	1.47 (1.16–1.87)	0.001
			No hypertension	Ref.	
Niiranen et al. [21••]	3614	CHD death	< 45	2.26 (1.75–2.93)	< 0.001
			45–54	2.18 (1.64–2.90)	n/a
			55–64	1.71 (1.26–2.32)	n/a
			≥ 65	1.36 (0.98–1.87)	0.07
			No hypertension	Ref.	
Suvila et al. [22]	2680	LVH	< 35	2.29 (1.36–3.86)	< 0.01
			35–44	1.67 (1.12–2.48)	< 0.05
			≥ 45	1.23 (0.74–2.03)	≥ 0.05
			No hypertension	Ref.	
Suvila et al. [22]	2680	LVDD	< 35	2.06 (1.04–4.05)	< 0.05
			35–44	1.59 (0.93–2.73)	≥ 0.05
			≥ 45	1.44 (0.75–2.79)	≥ 0.05
			No hypertension	Ref.	
Suvila et al. [22]	2680	Coronary calcification	< 35	2.94 (1.57–5.49)	< 0.001
			35–44	1.83 (1.10–3.05)	< 0.05
			≥ 45	1.41 (0.79–2.52)	≥ 0.05
			No hypertension	Ref.	
Suvila et al. [22]	2680	Albuminuria	< 35	1.12 (0.55–2.29)	≥ 0.05
			35–44	1.25 (0.74–2.09)	≥ 0.05
			≥ 45	0.62 (0.29–1.34)	≥ 0.05
			No hypertension	Ref.	

*CVD* cardiovascular disease; *CHD* coronary heart disease; *n/a* not available; *LVH* left ventricular hypertrophy; *LVDD* left ventricular diastolic dysfunction

^*^Myocardial infarction, stroke, congestive heart failure, or renal failure

Besides cardiovascular disease risk, two studies have also suggested that age of hypertension onset may be related to risk of developing dementia [24, 25•]. Gilsanz et al. examined the association between hypertension onset age, derived from two serial BP measurements, and incident dementia in 5646 women [24]. In this study, early-onset hypertension was not related to dementia, whereas mid-adulthood hypertension was associated with a 65% increase in risk of dementia. However, the risk estimates were elevated only among women. In another study by Corrada et al., performed in a highly selected cohort of 559 participants aged > 90 years, self-reported hypertension onset at 80–89 years was surprisingly related to a 42% lower risk of dementia, compared with those without hypertension [25•]. The finding was even more notable in those participants with hypertension onset age of 90+ years, as these participants had a 63% lower risk of dementia.

In more focused analyses on sex-specific differences in the CARDIA study, the impact of early-onset hypertension on cardiac effects seems to differ in men and women [31]. In that report, a prominent association of early-onset hypertension with left ventricular diastolic dysfunction (but not hypertrophy) was observed in women, in contrast to a prominent association of early-onset hypertension with increase in left ventricular hypertrophy (but not diastolic dysfunction) in men. It remains unclear if this finding explains the consistently observed female predominance of incident heart failure with preserved ejection fraction in later life, and whether it is a result of the sex differences in BP trajectories over the life course [43, 44].

Taking all available evidence together, it appears that early hypertension onset is associated with a considerable higher risk of cardiovascular disease than late-onset hypertension, most likely mainly representing the increased overall lifetime BP related to early-onset hypertension. Additional studies, however, are still needed to elucidate the potential sex-specific differences in the association between hypertension onset age and cardiovascular outcomes. In addition, the majority of the current evidence is based on case-cohort study designs, and prospective cohort studies in this domain are therefore needed. Finally, findings on the relation of early- versus late-onset hypertension and incident dementia are currently conflicting and scarce.

## Age of Hypertension Onset: How to Apply in Practice?

Considering the growing demand of more individualized treatment approaches along with increasing global burden of hypertension, a need for improved and feasible risk stratification methods exists. The potential advantages of assessing age of hypertension onset, instead of solely present, single-occasion BP, in clinical practice are listed in Table 2. Increasing evidence supports taking into consideration the cumulative lifetime burden of BP and other cardiovascular risk factors, instead of only single-occasion measurements, when assessing the risk of cardiovascular disease in patients [8, 45]. Even though several methods and indices have been previously proposed for assessing long-term exposure to high BP [8, 14••, 15, 16], their clinical use has remained limited due to their complexity which limits implementation into clinical practice. In contrast to these more elaborate indices, assessment of hypertension onset age could provide a feasible alternative for quantifying a patient’s lifetime BP load. It could be particularly used for improving cardiovascular risk assessment and BP control in younger patients, who often remain undiagnosed and/or untreated, but still carry a high lifetime cardiovascular disease risk [46, 47]. In addition to cardiovascular risk assessment, information on parental age of hypertension onset can be simultaneously used for assessing the risk of hypertension in offspring. Implementing age of hypertension onset assessment into clinical practice should therefore be used to target both hypertension therapy and screening efforts to those at highest risk.

**Table 2:** Potential advantages for assessing age of hypertension onset in clinical practice

Reference	Advantage of hypertension onset age assessment
Single-occasion BP measurement	Improved prediction of CVD outcomes
	Improved association with end-organ damage
	Allows for estimation of hypertension heritability
	Represents long-term BP exposure
Other indices of long-term BP exposure	Improved feasibility in clinical use
	Serial BP measurements not needed
	Can be defined using self-report
	Allows for estimation of hypertension heritability

*BP* blood pressure; *CVD* cardiovascular disease

Even though most of the current evidence on the association between age of hypertension onset and cardiovascular risk is based on objective BP measurements, some studies have also successfully used self-report for determining hypertension onset age [20•, 24, 25•, 26, 27, 29]. Considering the challenges related to accessing objective BP data from historical medical records from various healthcare providers, self-reported information on hypertension onset will most likely be the most relevant source of information in clinical practice. Physicians should therefore document the hypertension onset age of their newly diagnosed patients and their patients’ parents to improve hypertension management.

## Conclusions

Despite the varying methods used in different studies, current research indicates that early-onset hypertension has a stronger genetic component and is more strongly associated with cardiovascular outcomes compared with late-onset hypertension. However, the level of evidence on the pathogenesis, correlates, and prognosis of early versus late-onset hypertension still remains low, and several aspects in this domain require further study. First, established definitions for age of hypertension onset and early-onset hypertension need to be defined through expert consensus. Second, the genetic and clinical factors that predispose to early onset of hypertension and its transmission through generations need to be studied in detail. Third, most of the evidence on hypertension onset age as a risk factor for health outcomes comes from case-control studies with only cardiovascular outcomes. Additional prospective cohort studies with a wide range of clinical outcomes, such as dementia [24, 25•], are therefore needed. Fourth, a limited number of studies have suggested that sex-specific differences in the development and prognosis of early-onset hypertension exist. These differences need to therefore be studied in larger samples and in patients with various stages of cardiovascular disease.

The current evidence on hypertension onset age is still based on data from a limited number of observational studies. Thus, no definite evidence-based recommendations on its use in clinical practice can be made. Experimental studies that randomize patients to receive various therapies based on hypertension onset age could elucidate which treatment methods are the most effective for various age of onset groups. With accumulating evidence, assessment of hypertension onset age could be used as a feasible method for improving the risk stratification and personalization of therapy in hypertensive patients.
